# Mental Disorders, Musculoskeletal Disorders and Income-Driven Patterns: Evidence from the Global Burden of Disease Study 2017

**DOI:** 10.3390/jcm9072189

**Published:** 2020-07-10

**Authors:** Stefanos Tyrovolas, Victoria Moneta, Iago Giné Vázquez, Ai Koyanagi, Adel S. Abduljabbar, Josep Maria Haro

**Affiliations:** 1 Parc Sanitari Sant Joan de Déu, Universitat de Barcelona, Fundació Sant Joan de Déu, Dr Antoni Pujades, 42, Sant Boi de Llobregat, 08830 Barcelona, Spain; s.tyrovolas@pssjd.org (S.T.); mvictoria.moneta@pssjd.org (V.M.); i.gine@pssjd.org (I.G.V.); a.koyanagi@pssjd.org (A.K.); 2 Instituto de Salud Carlos III, Centro de Investigación Biomédica en Red de Salud Mental, CIBERSAM, Monforte de Lemos 3–5, Pabellón 11, 28029 Madrid, Spain; 3 ICREA, Pg. Lluis Companys 23, 08010 Barcelona, Spain; 4 Department of Psychology, King Saud University, Riyadh 11451, Saudi Arabia; abduljabbar@KSU.EDU.SA

**Keywords:** mental disorders, musculoskeletal disorders, DALYs, global

## Abstract

Background: The aim of the present study was to use the extensive Global Burden of Diseases, Injuries, and Risk Factors Study (GBD) database from 1990–2017 to evaluate the levels and temporal correlation trends between disability adjusted life years (DALYs) attributed to musculoskeletal (MSK) disorders, all mental disorders collectively and by mental disorder sub-category. Methods: We utilized results of the GBD 2017 to describe the correlation patterns between DALYs due to MSK disorders, mental disorders and other diseases among 195 countries. Mixed model analysis was also applied. Results: A consistent relation was reported between age-adjusted DALYs attributed to MSK and mental disorders (in total) among the 195 countries, in both sexes, for 1990 to 2017 (1990 *Rho* = 0.487; 2017 *Rho* = 0.439 *p* < 0.05). Distinct regional and gender correlation patterns between age-adjusted DALYs due to MSK and mental disorders were reported. No correlation was reported between DALYs due to MSK and all mental disorders collectively, among Low- or Middle-income countries. However, in High-income countries (HICs), the correlation was strong and consistent between 1990 and 2017 (1990 *Rho* = 0.735; 2017 *Rho* = 0.727, *p* < 0.05). Conclusions: The reported correlation patterns call for targeted preventive strategies and intervention policies for mental and MSK disorders internationally. Special attention is needed among HICs.

## 1. Introduction

Recent evidence from 63 different countries has shown that 29% of the population suffer from a mental disorder at least once during their lifetime [1]. Recently, data from the Global Burden of Disease (GBD) 2017 study reported that, globally, the age-standardized disability adjusted life years (DALYs) burden attributed to mental disorders for 2017 was 1606.76 DALYs per 100,000 (https://vizhub.healthdata.org/gbd-compare/), accounting for 4.89% of DALYs due to all health conditions, with mental disorders ranking 6th in terms of DALYs worldwide. 

Mental disorders have been associated with musculoskeletal (MSK) disorders [2,3,4,5], which also are among the largest contributors of disability globally. According the GBD 2017 data, MSK disorders ranked 5th in terms of DALYs worldwide for both sexes (https://vizhub.healthdata.org/gbd-compare/). MSK disorders include, among others, low back pain, gout, osteoarthritis and rheumatoid arthritis and are related with a variety of environmental and psychosocial factors (e.g., depression). Musculoskeletal impairments and mental disorders are both severe and persistent conditions causing low levels of self-perceived health and quality of life [6,7]. In addition, co-existence of both disorders increases the difficulty of their treatment [8,9]. Even though mortality rates of mental and MSK disorders are low [10], both of these disorders cause a great burden in terms of disability and distress. For example, MSK disorders increase physical limitation, cluster with mental disorders and raise healthcare expenditure [11]. Furthermore, there exists a complex relationship between MSK disorders and mental disorders where both conditions can mutually influence each other and create a loop, eventually leading to the aggravation of both conditions [12].

While MSK and mental disorder burden is analogous to that of cardiovascular diseases, to date, there are no global epidemiological data analyzing the relation among these disorders [13]. Specifically, although studies from some limited geographical regions do exist [14], the majority of these studies are small and do not always have the power to generalize their evidence at the country or cross-country level, and thus, translation into health policy and practice remain limited [12]. One recent study by Stubbs and colleagues [15] analyzed data from the World Health Survey on arthritis and mental health comparing 43 diverse settings. However, that study focused only on the relation between arthritis (a sub-category of MSK disorders) and a group of mental disorders (depression, psychosis, anxiety, sleep disturbances and stress). Furthermore, while the 2000–2010 bone and joint decade reported for the first time the burden of MSK disorders among various geographies, no information was shown regarding its mutual relation with mental health [16]. Thus, further evidence from a global perspective on the association between MSK and mental disorders is necessary. In particular, studies that include countries of different income levels are important. For example, impaired MSK health linked with mental disorders can lead to profound consequences on the population’s ability to participate in community’s activities, and lead to social isolation [12], especially in Low-income (LICs) and Middle-income countries (MICs) [11]. Furthermore, although information is still limited, there is some evidence that suggests that the relation between impaired mental health and MSK disorders would be stronger in higher income countries than among lower income countries [15]. Finally, studies that examine the change in the patterns of correlation among mental and MSK disorders over time is lacking.

Thus, the aim of the present study was to use the extensive GBD database from 1990 to 2017 to evaluate geographical, socio-demographic and temporal trends of the correlation between MSK and mental disorders. First, we hypothesized that mental and MSK disorders and their subcategories (expressed as age-adjusted DALYs estimates) will be correlated consistently between 1990 and 2017 among 195 countries. Secondly, we hypothesized that this correlation will be different by sex and by country-income levels (High-, Middle- and Low-income). In terms of comparisons, the correlation between YLDs due to MSK disorders and other disorders, such as cardiovascular diseases, neoplasms, neurological disorders and injuries is also presented.

The GBD is among the few global health studies that apply standard design and procedures across all populations of the world (195 countries). To the best of our knowledge, except for this dataset, there are no other multi-country studies on MSK and mental disorders that allow comparisons by sex and between different settings and/or country-income levels.

## 2. Methods

### 2.1. Geography and Metrics

GBD 2017 quantified multiple measures of health loss for 359 causes and 84 risk factors for 195 countries and territories, from 1990 to 2017. For the present analysis, we compared all available 195 locations. Our comparisons are primarily for both sexes combined, by males and females and by country-income level. GBD results are available publicly for visualization (http://vizhub.healthdata.org/gbd-compare) and for download (http://ghdx.healthdata.org/gbd-results-tool).

The GBD study’s protocol has been approved by the research ethics board at the University of Washington (UW). The GBD shall be conducted in full compliance with UW policies and procedures, as well as applicable federal, state and local laws.

### 2.2. Mental Disorders and Musculoskeletal Disorders

As presented in Table 1, mental disorders were separated into 13 sub-categories, and musculoskeletal disorders were separated into 6 sub-categories for in-depth analysis, following GBD 2017 methodology. Mental disorders as a total estimate are reflected through the item total mental disorders (MDT). Epidemiologic estimations such as prevalence and incidence could be found through the Epi Visualization tool (https://vizhub.healthdata.org/epi/). Mental and MSK disorders sub-categories were defined based on specific GBD criteria. For example, in GBD, anxiety disorders were classified as all the experiences of intense fear and distress, typically in combination with other physiological symptoms [17]. More in detail, anxiety disorders were classified as those cases reaching a diagnostic threshold defined by the Diagnostic and Statistical Manual of Mental Disorders (DSM) or the WHO International Classification of Diseases (ICD-10) [17]. Excluded cases were anxiety disorders due to a general medical condition or a substance-induction. Moreover, other mental disorders were classified as an aggregate group of personality disorders (i.e., paranoid personality disorder, dependent personality disorder and others) [13,17]. The definition of rheumatoid arthritis for the GBD was based on the American College of Rheumatology criteria. For rheumatoid arthritis, the following ICD-10 codes were included (M05, M06 and M08) and also the following ICD-9 codes (714.0–714.9) [17]. Detailed definitions of all subcategories of MSK and mental disorders are provided in previous published GBD research [13,17].

In this study, we used specific GBD metrics. We compared DALYs rates across locations with age-standardization to control for age structure, and correlation analysis was conducted to examine specific interrelations between MSK and mental disorders (http://www.healthdata.org/terms-defined). Annualized rate of change (ARC%) was computed as natural log-transformed (final estimates/initial estimates) divided by the number of years and was used to compare trends among age-standardized DALYs. No specific ARC threshold was used to classify a negative or positive change. Detailed methods for the overall study and for specific causes and risks are available in GBD 2017 summary publications, [13,17] each of which is GATHER-compliant [18]. A brief description of methods (i.e., mortality and causes of death, years of life lost (YLLs)) used for deriving disability adjusted life-years (DALY) and years lived with disability (YLD) metrics included in the present analysis are provided below in parts 2.3 to 2.5.

### 2.3. Mortality and Causes of Death

Mortality estimation methods are described in detail elsewhere [10] but are briefly explained here since they are the first step to further estimate non-fatal health loss. All-cause mortality was estimated primarily from vital registration (VR) data, adjusted for completeness among all GBD locations. Spatiotemporal effects and covariates were used to extend estimates to 2017. The lowest observed risk of death for each age group in total populations of greater than 5 million were summed to construct a global standard life expectancy.

For the calculation of life expectancy at birth (LE), the existing GBD model life table system (which identifies a reference table of each location, year and sex, based on the nearest matches in the GBD empirical life table database) was used. Using this updated dataset, the LE estimated the life expectancy at birth for the all the available populations [19].

Cause-specific deaths and YLLs were estimated for underlying causes of mortality, ensuring that the sum of all specific causes is equal to all-cause mortality. Each death was assigned to a single cause. Vital registration and census death data were adjusted for incompleteness and misclassification (e.g., prostate cancer in a female); non-specific and intermediate codes (e.g., sepsis, heart failure, unknown causes) were redistributed using age-, sex- and geography-specific statistical redistribution methods prior to modelling. The most commonly used estimation method was cause of death ensemble modelling (CODEm). CODEm uses a train-test-test approach, first testing all combinations of selected country-level covariates and their relationship to in-sample data, then ranking those component models based on out-of-sample predictive validity to construct weighted ensembles. All ensemble models are again ranked on a second round of out-of-sample predictive validity to select a final model. Cause-specific fractions were multiplied by all-cause mortality estimates to calculate cause-specific deaths, then scaled with all other causes to match all-cause mortality. YLLs were calculated by multiplying age-specific deaths by global standard life expectancy at age of death.

For this analysis, age-standardized YLLs for MSK disorders and mental disorders, cardiovascular diseases, neoplasms, neurological disorders and injuries were estimated, for all locations, for both sexes combined as well as by males and females and by country-income level. Information on the YLLs due to cardiovascular diseases (CVDs) (including disorders in cardiomyopathy, stroke and other), neoplasms (i.e., stomach cancer), neurological disorders (clustering disorders such as Alzheimer’s, Parkinson) and injuries (grouping among others road injuries, unintentional injuries) definition is available in detail elsewhere [10,13].

### 2.4. Nonfatal Health Loss as Expressed by Years Lived with Disability (YLDs)

Epidemiologic data from systematic literature review, health surveys, surveillance systems, disease registries and hospital and claims databases were used to generate cause- and sequela-specific prevalence and incidence estimates using a variety of modelling approaches, of which Bayesian meta-regression compartmental modelling in DisMod-MR 2.1 was the most common [20]. Disability weights (DW) for each unique health state were reported in the last GBD cycle [21]. A microsimulation framework was then used to adjust for comorbidity, and YLDs for each cause were calculated by multiplying prevalence and corresponding disability weights (DW) for each sequela of each cause [21]. Again, for our purpose, the age-standardized YLDs for MSK disorders, mental disorders (all categories) cardiovascular diseases, neoplasms, neurological disorders and injuries were estimated during 1990 to 2017.

### 2.5. Disability-Adjusted Life-Years (DALYs)

The disability-adjusted life-years (DALYs) is a summary measure of health that was calculated for each age-sex-year-state-cause strata by summing the fatal (YLL) and nonfatal (YLD) components [13]. DALYs in this research work were estimated for the all aforementioned items of interest (i.e., mental disorders, MSK disorders).

### 2.6. Assessment of Socioeconomic Factors and Health System Performance

To further assess the effects of socioeconomic factors and health system performance on global and regional health metrics, we employed the Socio-demographic Index (SDI) and the Healthcare Access and Quality (HAQ) Index, respectively. SDI is a composite indicator that uses as components the country-level income per capita, average educational attainment among individuals over age 15 and total fertility rate among women under 25. The SDI ranges from 0 to 100 [13].

HAQ Index is a GBD composite metric that is based on comparative mortality rates for health-care-sensitive diseases and standardized to risk exposure level and is meant to quantify the overall performance of health systems. The HAQ Index ranges from 0 to 1 [22].

### 2.7. Statistical Analysis

#### 2.7.1. Correlation Analysis

Associations, primarily between age-standardized DALYs and YLDs for MSK and mental disorders (all categories) and secondarily between MSK disorders and cardiovascular diseases, neoplasms, neurological disorders and injuries (age-standardized DALYs and YLDs), were tested by the Spearman’s rho coefficients. The associations were tested for the years 1990, 1995, 2000, 2005, 2010, 2015 and 2017 among all 195 countries, for both sexes, by males and females and by country-income level based on the World Bank classification (high-income countries (HICs), lower and upper middle-income countries (LMICs), low-income countries (LICs)) [23]. All *p*-values are based on two-sided tests. *p* value ≤ 0.05 was considered as significant. We followed previously reported criteria to classify a correlation as weak (0.3≤), moderate (0.4–0.6), and strong (≥0.7) (coefficients are presented as absolute values) [24]. When a correlation was significant across all time points and in the same direction, it was considered as “consistent”. Consistency in the abovementioned correlation was additionally tested using longitudinal linear mixed model analysis, as reported in detail below.

#### 2.7.2. Linear Mixed Model Analysis

Mixed-effect multilevel linear regression models were carried out to assess whether MDT (outcome) and MSK disorders (independent variable) (as DALYs) were related between 1990 and 2017, after adjustment for various factors. Specifically, the mixed-effect multilevel model [25] was conducted to assess the effects of time (1990–2017) and MSK disorders on mental disorders, adjusting for the effect of SDI, SDIxHAQ index (Model I) and adjusting for the effect of SDI, SDIxHAQ index, CVDs, neurological disorders, neoplasms and injuries (Model II). Maximum likelihood estimation was used in the multilevel analysis; time was considered as the first level of analysis while the countries were considered as the level (2nd level) where the observations of the repeated measures were aggregated.

Correlation analyses were performed with the Statistical Package for Social Sciences version 22 (SPSS Inc., Chicago, IL, USA) and linear mixed model analysis with the R package lme4.

## 3. Results

### 3.1. Trends in DALYs Due to Mental and MSK Disorders and Annualized Rates of Change (ARC) between 1990 and 2017

**Figure 1:**
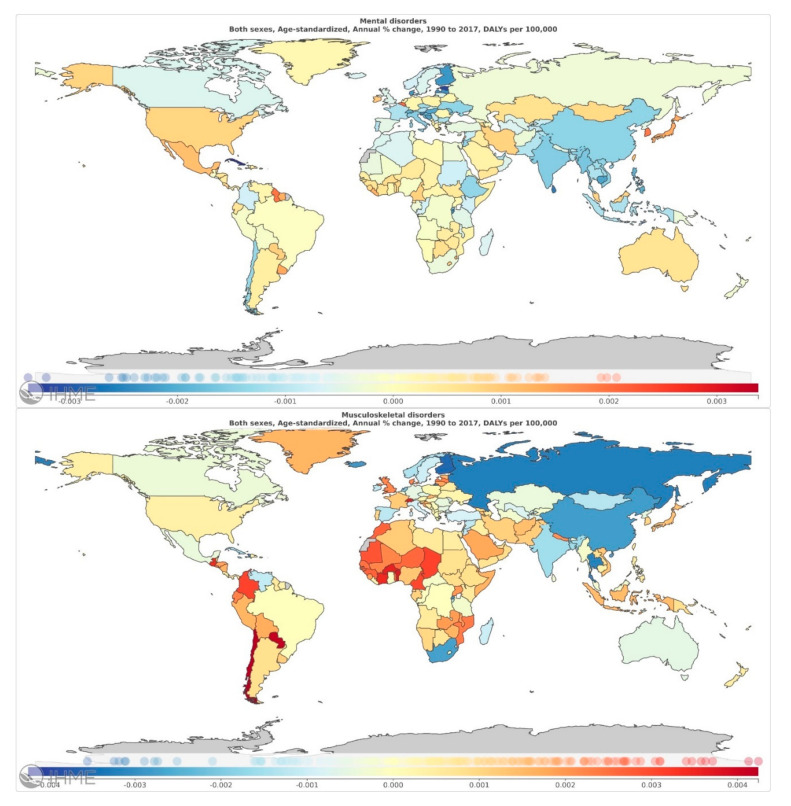
Annualized rate of change (ARC%) of age-standardized total mental disorders (MDT) and musculoskeletal DALYs rate, in both sexes, between 1990 and 2017. Map was generated using the GBD visualization tool provided online by the Institute of Health Metrics and Evaluation (https://vizhub.healthdata.org/gbd-compare/)

Analysis of the ARC% of age-standardized DALYs attributed to mental and MSK disorders, between 1990 and 2017, in both sexes and by country is presented in Supplementary Table S1. There were 75 countries out of 195 with a positive ARC% of DALYs attributed to mental disorders between 1990 and 2017, while for the corresponding number of countries with positive ARC for MSK disorders was 128. The countries with the highest age standardized mental disorder DALYs rates were Belgium (0.207% ARC), South Korea (0.193% ARC) and Guyana (0.198% ARC). In addition, those with the highest age standardized MSK disorder DALYs rates were Chile (0.424% ARC), Paraguay (0.413% ARC) and Benin (0.371% ARC). Results for all the countries are shown in detail in Figure 1. 

### 3.2. Correlation Trends in DALYs Attributed to Mental and MSK Disorders between 1990 and 2017

**Figure 2:**
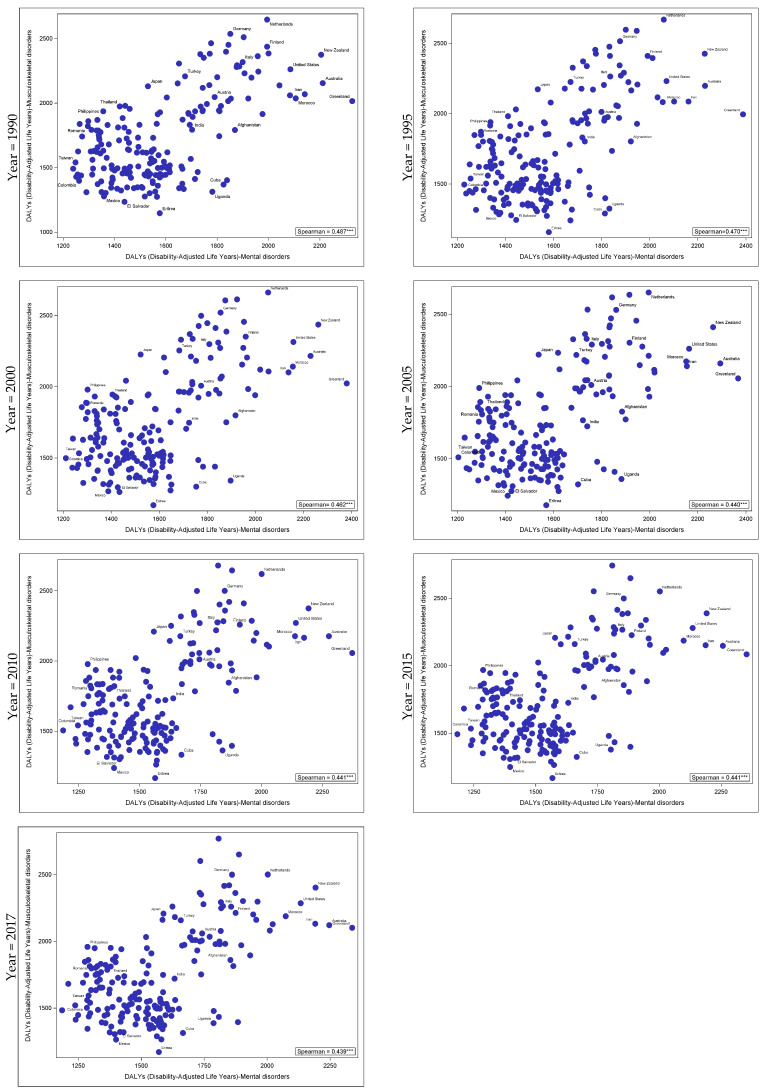
Correlation between age-standardized musculoskeletal and total mental disorders (MDT) DALYs among 195 countries, for both sexes, between 1990 and 2017. *** *p* < 0.0001.

Table 2 and Supplementary Table S2 presents the correlation coefficients between MSK disorders, mental disorders and other health outcomes with age-standardized DALYs for 195 countries, in both sexes, between 1990 and 2017. A consistent and moderate positive correlation between DALYs attributed to MSK and mental disorders (MDT) was observed from 1990 to 2017 (Table 2: 1990 *Rho* = 0.487; 2017 *Rho* = 0.439 *p* < 0.05). These correlations at 5-year intervals are also available in Figure 2. When focusing on the sub-categories of mental disorders, a consistent and moderate positive correlation between DALYs attributed to MSK disorders, anxiety (Table 2: 1990 *Rho* = 0.548; 2017 *Rho* = 0.503 *p* < 0.05), eating disorders (Table 2: 1990 *Rho* = 0.461; 2017 *Rho* = 0.451 *p* < 0.05), the category of other mental disorders and schizophrenia (*p* < 0.05) was shown. A weak consistent positive correlation was observed between DALYs attributed to dysthymia and bipolar, autism (*p* < 0.05) and MSK disorders, while no association was shown with depressive disorders or major depressive disorders (MDD). In addition, a consistent inverse correlation was shown for attention-deficit/hyperactivity disorders and MSK conditions (Table 2: 1990 *Rho* = −0.303; 2017 *Rho* = −0.305, *p* < 0.05). No association was found between DALYs attributed to MSK disorders and major depressive disorders, conduct disorders, idiopathic developmental intellectual disability or with neoplasms and neurological disorders. Consistent weak to moderate inverse relations were shown between DALYs attributed to musculoskeletal disorders and cardiovascular diseases as well as with injuries.

In terms of the sub-categories of MSK disorders, a consistent and moderate positive correlation between DALYs attributed to MDT and rheumatoid arthritis (Supplementary Table S2: 1990 *Rho* = 0.484; 2017 *Rho* = 0.541 *p* < 0.05), osteoarthritis (Supplementary Table S2: 1990 *Rho* = 0.446; 2017 *Rho* = 0.420 *p* < 0.05), low back pain (*p* < 0.05) and the sub-category of gout (Supplementary Table S2: 1990 *Rho* = 0.353; 2017 *Rho* = 0.366 *p* < 0.05) was shown.

**Figure 3:**
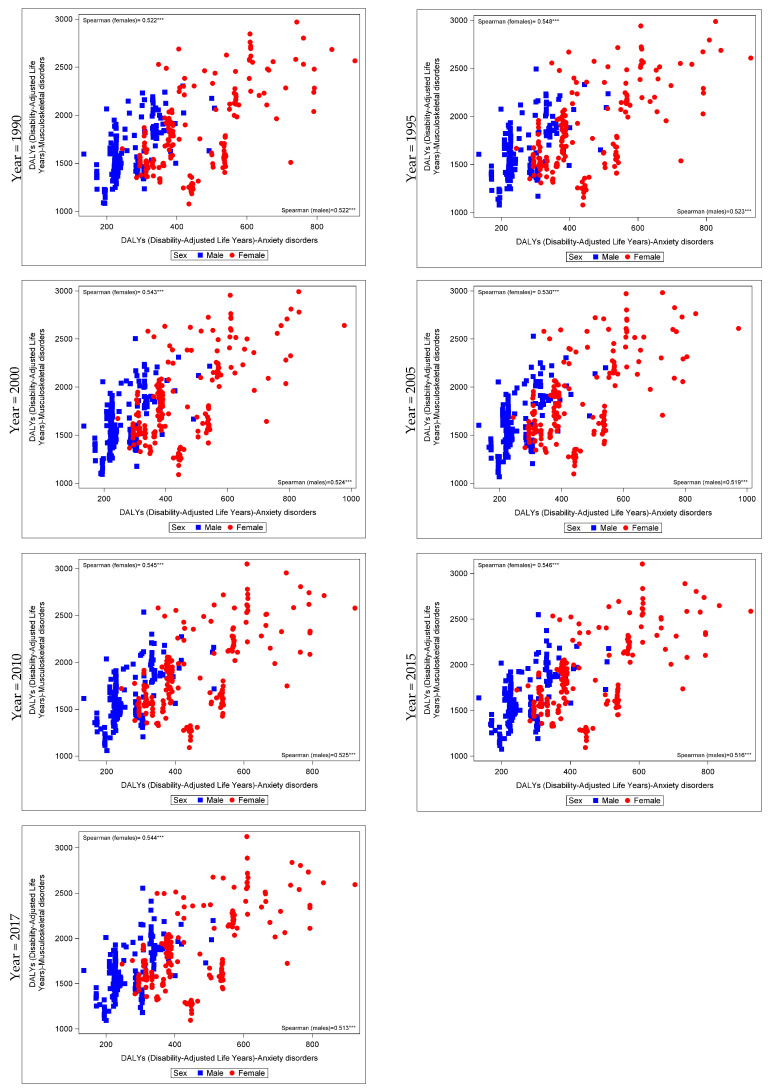
Correlation between age-standardized musculoskeletal and anxiety disorders DALYs among 195 countries, analyzed by gender, between 1990 and 2017. *** *p* < 0.0001.

Correlation coefficients between MSK disorders, mental disorders and age standardized DALYs of other health outcomes for 195 countries, in males and females, between 1990 and 2017 are shown in Table 3 and Supplementary Table S3. Stratified analysis by gender revealed similar trends in the correlations between DALYs attributed to MSK and mental disorders for males and females, but of different magnitude. Analyzing the relation between MSK disorders and all subcategories of mental disorders, DALYs due to MSK disorders were related moderately to strongly, mostly with any mental disorder, anxiety and the special category of all mental disorders except anxiety and depressive disorders (*p* < 0.05). Between 1990 and 2017, MSK disorders were inversely related with conduct disorders in females but positively related in males (although a weak correlation was observed). Moreover, only in males, autism disorders were positively related with MSK disorders (*p* < 0.05). Similar trends were shown for analyses between MDT, and all subcategories of MSK disorders (Supplementary Table S3). Correlation of DALYs attributed to anxiety and MSK disorders, among females and males is shown in Figure 3 (*p* < 0.05).

In order to further explore whether mental and MSK disorders are related across time and go beyond univariate correlation analysis, a mixed-effect multilevel regression analysis was conducted, adjusting for various factors and taking into account the time effect. Specifically, in Model I, after adjusting for SDI, SDIxHAQ index and time (year), MSK disorders were positively related with mental disorders (MDT) for both sexes (Coef: 0.137, S.E:0.02, *p* < 0.001). In Model II, after adjusting for the abovementioned covariates plus CVDs, neoplasms, neurological disorders and injuries, a similar positive relation between mental and MSK disorders was found (Coef: 0.126, S.E: 0.02, *p* < 0.001) (data shown only in text).

### 3.3. Correlation Trends in DALYs Attributed to Mental and MSK Disorders between 1990 and 2017, by Country-Income Level

**Figure 4:**
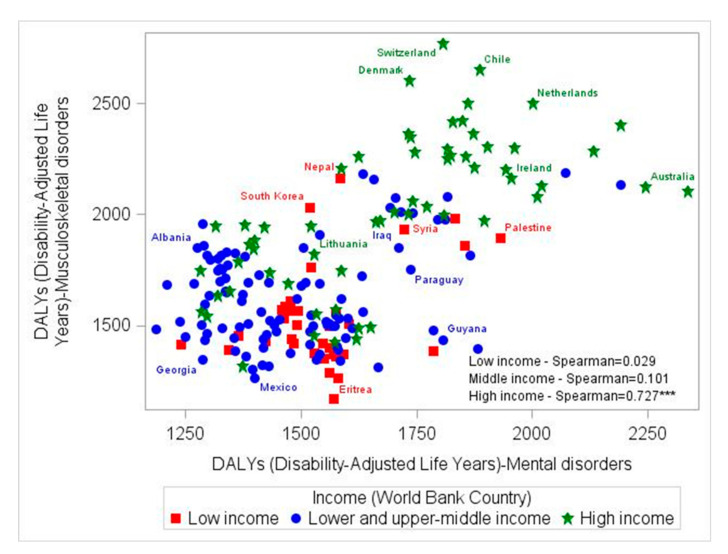
Correlation between age-standardized musculoskeletal and total mental disorders (MDT) DALYs among 195 countries, analyzed by income level, for 2017. DALYs: Disability-adjusted life-years. *** *p* < 0.0001.

A comparison by country-income level of the correlation trends between age-standardized DALYs attributed to MSK and mental disorders from 1990 to 2017 helps to identify different regional patterns (Table 4 and Supplementary Table S4). It was shown that there was no correlation between DALYs due to MSK and mental disorders (MDT) among LICs and MICs. However, in HICs, the aforementioned correlation was strong and consistent between 1990 and 2017 (Table 3: 1990 = 0.735; 2017 *Rho* = 0.727, *p* < 0.05). Figure 4 illustrates the aforementioned association by country-income level only for 2017. Similar patterns were obtained between DALYs attributed to MSK disorders and depressive disorders, dysthymia and other mental disorders (correlations presented only among HICs). DALYs attributed to anxiety and MSK conditions were related consistently between 1990 to 2017 among LMICs and HICs, while idiopathic developmental intellectual disability was correlated with musculoskeletal disorders only among LMICs. The special category of all mental disorders except anxiety and depressive disorders (aMeAD) showed a positive correlation for 1990 to 2017 among LICs, while a consistent strong correlation with MSK disorders was showed among HICs. Interestingly, dysthymia, eating disorders and autism presented positive and inverse correlations with MSK conditions in LICs and LMICs but a positive one among HICs (*p* < 0.05). Schizophrenia was moderately correlated with MSK disorders among LICs but this relation was weak among LMICs and HICs (*p* < 0.05). No correlation between DALYs due to conduct and MSK disorders was observed among all regions (HICs and LMICs).

Analyzing the relation between mental disorders in MDT and all subcategories of MSK disorders, taking into account each country’s income for 2017, it was found that DALYs due to mental disorders were related moderately to strongly, with rheumatoid arthritis among all regions (*p* < 0.05). However, the rest of the MSK sub-categories (osteoarthritis, low back pain, neck pain and gout) were associated moderately with mental disorders (MDT) only among HICs. No relation was observed among LICs and MICs (Supplementary Table S4).

### 3.4. Correlation Trends in YLDs Attributed to Mental and MSK Disorders between 1990 and 2017, in Both Sexes, by Males and Females and by Country-Income Level

Correlation coefficient trends between age-standardized YLDs attributed to mental and MSK disorders in both sexes by males and females and by country-income level followed similar patterns with the previously presented DALYs (Supplementary Tables S5–S7). However, in both sexes, the age-standardized YLD correlation coefficients were consistent and inclining trends between MSK disorders and almost all the sub-categories of mental disorders were observed (*p* < 0.05). The YLDs attributed to mental and MSK disorders correlation coefficients when analyzed by gender and by country-income level showed a stable and consistent pattern comparable with the previous one reported for DALYs.

## 4. Discussion

The present work analyzed the geographical and temporal burden of mental and MSK disorders and their correlation between 1990 and 2017. We found that 75 countries around the world had concomitant positive ARC% for mental and MSK disorders during 1990–2017. Second, a consistent relation between age-adjusted DALYs attributed to MSK and mental disorders (as reflected by MDT), among the 195 GBD countries, in both sexes, for 1990 to 2017 was shown. This finding was also confirmed when applying mixed models analysis adjusting for various confounders. Anxiety, eating disorders, all mental disorders except anxiety and depressive disorders and other mental disorders had the strongest correlation with MSK disorders, between 1990 and 2017, while no relation was observed with depressive disorders. In addition, MDT was moderately related with rheumatoid arthritis, osteoarthritis, low back pain, neck pain and gout. Third, analysis stratified by gender showed that the aforementioned relations followed similar correlation patterns among males and females between 1990 and 2017. However, DALYs attributed to dysthymia and eating disorders reported higher correlation coefficients with DALYs due to MSK disorders in females. Fourth, when country income was taken into account, there was a clear correlation pattern between DALYs due to MSK disorders and all the categories of mental disorders across HICs. A similar and consistent pattern was shown between DALYs due to MDT and all the categories of MSK disorders, across HICs, while a positive correlation with rheumatoid arthritis was observed across all regions.

It is well known that musculoskeletal disorders coexist with chronic conditions [9] and normally formulate part of multimorbidity clusters (i.e., diabetes mellitus, heart disease, respiratory disorders, cancer) [12]. In addition, they share many common risk factors between each other, such as physical inactivity, obesity, smoking habits and nutrition of low quality [11]. At the population level, according to our analysis, age-standardized YLDs due to MSK disorders were related with YLDs due to neoplasms, injuries, neurological disorders and CVDs (Supplementary Table S5), consistently between 1990 and 2017, but not with YLDs attributed to mental disorders. Interestingly, among LMICs, YLDs attributed to CVD were positively related with YLDs due to MSK disorders, while among HICs, this relation was reversed. This could be a result of different data quality between HICs and LMICs.

As aforementioned, MSK and mental health have a complex interrelation that can potentially exacerbate or cause the other [12]. The main pathway between the association of mental and musculoskeletal disorders is based on the same cerebral-processing channels that are mutually related with serotonin metabolism [26]. Our analysis showed a positive and consistent correlation pattern between age-standardized DALYs due to MSK and mental disorders in total. Recent studies have shown a strong association between various subcategories of mental disorders, such as anxiety and mood disorders as well as with substance use [12]. When MSK disorders are accompanied by pain (as often happens), comorbid depression has been reported in some cases, although not all [27]. Additional pathways through which MSK disorders could cause mental health problems include functional impairment, loss of work, sexual difficulties, independence loss and social withdrawal and isolation [12]. Our analysis reported a positive and consistent correlation pattern between DALYs due to MSK disorders, anxiety and various mental disorders subcategories, but no relation emerged from our data between MSK and depressive disorders or MDD [27]. This finding is not in line with the literature and may be attributed to the nature of the current analysis, which was based on aggregated data rather than individual data. However, it could also be possible that some of the previous findings could be influenced by the co-morbidity of depressive and anxiety disorders. Local epidemiological studies have shown that depressive symptomatology is related with musculoskeletal disorders and pain [3,28]. Contrary to what may be expected, ADHD was inversely related with age-adjusted DALYs due to MSK disorders [29,30]. The latter could be attributed to the application of univariate correlation analysis that did not take into account the effect of other confounders such as ADHD medication and other medical conditions. The consistent and positive associations between MSK disorders and various categories of mental disorders found in our analysis call for enhancements of preventive policies and mutual intervention strategies (between MSK disorders and anxiety disorders, major depressive disorders, dysthymia) at specific population groups.

Additional analysis by gender showed similar trends in the correlation patterns between age-adjusted DALYs due to MSK and mental disorders (MDT), although the coefficients were attenuated in males. This could be attributed to the fact that mental disorders (i.e., anxiety, depression) and MSK are more pronounced in females, as has been reported in the literature [31], but also this could be due to women and men experiencing different types of musculoskeletal disorders. Specifically, it has been reported that women tend to suffer more from fibromyalgia, temporomandibular disorders and neck pain, while men tend to have more osteoarthritis. The main pathway for the aforementioned is that men and women also have a different composition of muscle mass, with women having more type II fibers and men more than type I fibers [32]. Moreover, the mechanism of psychological disorders, inflammation and musculoskeletal disorders may also explain the abovementioned findings [33,34], a pathway that is more pronounced among females [35,36]. In addition, MSK and autism disorders were related only among males. Autism is frequently accompanied by neuromuscular and musculoskeletal manifestations, a fact that in conjunction with the much higher prevalence of the disorder among male population could explain our findings [37]. In addition to this finding, a distinct correlation pattern between MSK and conduct disorders among genders was observed. While gender differences have been reported in conduct disorders in the past [38], this is the first time that such a finding is reported at population level. Future studies, especially with individual data and longitudinal analysis, are warranted to assess whether this relation can be replicated. The aforementioned highlights the immediate need for targeted public health policies that take into account gender differences.

Some distinct correlation trends between MSK and mental disorders were found in HICs and LMICs (i.e., MSK were not related with ADHD, IDID). Interestingly, no relation was evident from 1990 to 2017, between MSK and mental disorders (in total -MDT-), depressive disorders and MDD in LMICs, while moderate/strong correlations were observed in HICs. Furthermore, although no relation between age-adjusted DALYs due to anxiety and MSK was shown among LICs, moderate positive correlations were found in MICs and strong correlations in HICs. Based on the aforementioned findings, it should be noted that mental and MSK disorders are relatively highly prevalent among HICs [1,13], a fact that could lead to an increased correlation between them even though they may be independent, since the overlap is bound to increase with higher prevalence of one of the two disorders. Additionally, moderate correlations were shown for MDT and all MSK disorder categories (except other MSK disorders) as defined by pain (i.e., low back pain, neck pain) or inflammation (i.e., gout) only among HICs. Rheumatoid arthritis was consistently related with MDT between 1990 and 2017 across all regions. These results are in line with those of individual studies conducted in LMICs and HICs [15,39,40]. However, our study is the first to report the diverse geographical/regional correlation patterns between mental and MSK disorders, using population-based data from the GBD 1990–2017 study.

Currently, individuals around the world and mostly among high-income regions are experiencing high burden of disability due to mental and MSK disorders [12,13,41], as shown by our analysis at the population level, which tested the association between MSK disorders and all mental health categories and vice versa. This income driven association could be explained by the different nature of ageing and disease burden between HICs and LMICs. Specifically, in HICs, the population has grown considerably older, and disease burden is characterized more by disability (caused mostly by MSK and mental disorders, as well as injuries) rather than by premature mortality. However, LMICs are facing a different “nature” of disease burden (i.e., high premature mortality due to communicable diseases) and risk factors [13,17]. Supporting the later, across 195 GBD countries, life expectancy increases with increasing country-income level between 1990 to 2017, for both sexes (1990: LICs = 54.1 ± 7.56 years of LE, MICs = 66.0 ± 6.38 years of LE, HICs = 74.0±2.91 years of LE; 2017: LICs = 64.6 + 5.54 years of LE, MICs = 71.8 ± 5.29 years of LE, HICs = 79.3 ± 3.32 years of LE). This difference could also be explained by the different diagnostic criteria used in LMICs and HICs for both MSK and mental disorders, as well as cultural/occupational factors (e.g., MSK disorders due to accumulated sedentary office work and low physical activity in HICs, in comparison to MSK disorders due to intense physical labor in LICs). In addition to the aforementioned, while it is well known that MSK disorders can negatively affect independent living and consequently lead to isolation and psychological distress, MSK disorders lack recognition among health stakeholders [12]. These facts, along with the previously discussed distinct regional correlation patterns, indicate the need for specific investments for prevention and intervention plans with a joint impact on mental and MSK disorders following different strategies for HICs and LMICs. Countries with greater DALYs due mental disorders also reported greater DALYs due to MSK, a finding that calls for countries to address both conditions with joint health policies. Sustainable and targeted prevention strategies in LMICs could be helpful to avoid a similar pattern of high burden of MSK and mental disorders that HICs currently face [42]. Furthermore, future studies should evaluate the effectiveness of specific interventions and other cost-effective actions to decrease disability due to mental and MSK disorders.

## 5. Strengths and Limitations

The present study has several strengths. To the best of our knowledge, this is the first global study to evaluate the association between age-standardized DALYs attributed to MSK and any mental disorders, as well as by mental and MSK disorder categories using the GBD 2017 platform with standardized data that allow global comparisons of different settings, which has not been done previously. These results could help policy makers develop prevention, intervention and treatment programs for MSK and mental disorders. However, the present analysis shares the limitations of other GBD studies, mostly the challenges of capturing all sources of uncertainty, lags in data availability, variation in coding practices (i.e., possible regional differences in assessment of mental and MSK disorders), DALYs construct and other biases and limitations of existing analytical tools, which may not fully capture temporal trends in mortality, incidence and prevalence [13]. Moreover, the GBD 2017 study assumes isolated presence of each disorder at the population level, meaning that the diseases may not necessarily co-exist in the same individual. Finally, the univariate correlation analysis between DALYs due to mental and MSK disorders does not take into account the confounding effect of additional factors; thus, the results should be interpreted with caution. However, the results of the additional mixed effects analysis were in line with the correlation analysis.

## 6. Conclusions

We found a consistent and positive association between age-adjusted DALYs of mental and MSK disorders from 1990 to 2017, in 195 countries. Among mental disorder sub-categories, anxiety showed the highest correlation with MSK disorders. This association was more pronounced among females and in HICs. Depressive disorders were related with MSK disorders only among HICs. Among MSK disorders, rheumatoid arthritis was related with MDT across all regions. The gender-dependent and regional correlations call for implementation of context- or population-specific direct and simultaneous preventive policies for both mental and MSK disorders. In particular, addressing these conditions in the context of HICs would be important. Policymakers should consider a health care system where mental and musculoskeletal healthcare services could address patient’s physio-psychosocial aspects jointly.

## Figures and Tables

**Table 1:** Mental and musculoskeletal disorders’ subcategories and definition.

Mental Disorders	Definition/Estimation	Musculoskeletal Disorders	Definition/Estimation
aMeAD	the sum of DALYs attributed to total mental disorders (MDT) minus DALYs attributed to anxiety and depressive disorders	Rheumatoid arthritis	GBD original definition
Depressive disorders	the sum of DALYs for major depressive disorders and dysthymia	Osteoarthritis	GBD original definition
Major depressive disorders	GBD original definition	Low back pain disorders	GBD original definition
Anxiety disorders	GBD original definition	Neck pain disorders	GBD original definition
Attention deficit/hyperactivity disorder	GBD original definition	Gout disorders	GBD original definition
Idiopathic developmental intellectual disability	GBD original definition	Other musculoskeletal disorders	GBD original definition
Anorexia or bulimia nervosa (eating disorders)	the sum of DALYs attributed to anorexia or bulimia nervosa	-	-
Other mental disorders	GBD original definition	-	-
Schizophrenia	GBD original definition	-	-
Dysthymia	GBD original definition	-	-
Bipolar disorders	GBD original definition	-	-
Autism disorders	GBD original definition	-	-
Conduct disorders	GBD original definition	-	-

aMeAD: All Mental except Anxiety and Depressive disorders; DALYs: disability-adjusted life-years; Global Burden of Diseases, Injuries, and Risk Factors Study (GBD) original definition: Detailed definitions of all subcategories of MSK and mental disorders are provided in previous published GBD research appendices [13,17].

**Table 2:** Spearman’s correlation coefficients between age-standardized DALYs attributed to musculoskeletal disorders and age-standardized DALYs attributed to various disorders, among 195 countries, in both sexes, for 1990 to 2017.

		Other Disorders				Mental Disorders Sub-Categories													
	Year	Neoplasms	CVD	NeuroD	Injuries	MDT	aMeAD	Depressive Disorders	MDD	Dysthymia	Bipolar	Anxiety	EatingD	Autism Disorders	ADHD	Conduct Disorder	IDID	OMD	SZA
MSK	1990	0.105	−0.089	0.038	−0.319	0.487	0.630	0.144	0.051	0.339	0.276	0.548	0.461	0.130	−0.303	−0.002	0.073	0.470	0.602
	1995	0.054	−0.176	0.002	−0.391	0.470	0.613	0.120	0.038	0.320	0.286	0.530	0.472	0.136	−0.301	0.014	0.042	0.495	0.582
	2000	0.009	−0.239	−0.002	−0.381	0.462	0.589	0.126	0.044	0.302	0.284	0.522	0.477	0.149	−0.280	0.010	0.009	0.488	0.569
	2005	−0.008	−0.255	0.028	−0.390	0.440	0.573	0.114	0.033	0.309	0.272	0.511	0.466	0.154	−0.290	0.037	0.023	0.474	0.559
	2010	−0.045	−0.277	0.056	−0.444	0.441	0.570	0.108	0.028	0.315	0.269	0.513	0.462	0.151	−0.297	0.023	0.038	0.456	0.549
	2015	−0.124	−0.303	0.047	−0.417	0.441	0.565	0.104	0.023	0.322	0.272	0.506	0.457	0.159	−0.304	0.025	0.076	0.452	0.539
	2017	−0.134	−0.307	0.041	−0.442	0.439	0.563	0.104	0.017	0.320	0.272	0.503	0.451	0.166	−0.305	0.023	0.090	0.448	0.533

All significant correlations (*p* < 0.05) are in red. DALYs: Disability-adjusted life-years; CVD: Cardiovascular disorders; NeuroD: Neurological Disorders; MDT: Mental Disorders Total; aMeAD: All Mental except Anxiety and Depressive disorders; MDD: Major Depressive Disorders; ADHD: Attention deficit/hyperactivity disorder; IDID: Idiopathic Developmental Intellectual disability; EatingD: Anorexia or Bulimia nervosa; OMD; Other Mental Disorders; SZA: Schizophrenia; MSK: Musculoskeletal Disorders. MSK is the sum of DALYs due to rheumatoid arthritis, osteoarthritis, low back pain, neck pain, gout and other MSK disorders.

**Table 3:** Spearman’s correlation coefficients between age standardized DALYs attributed to musculoskeletal disorders and age-standardized DALYs attributed to various disorders, among 195 countries, in males and females, for 1990 to 2017.

			Other Disorders				Mental Disorders Sub-Categories													
	Year	Gender	Neoplasms	CVD	NeuroD	Injuries	MDT	aMeAD	Depressive Disorders	MDD	Dysthymia	Bipolar	Anxiety	EatingD	Autism Disorders	ADHD	Conduct Disorder	IDID	OMD	SZA
MSK	1990	Females	−0.112	−0.279	0.169	−0.301	0.448	0.646	0.175	0.058	0.442	0.329	0.552	0.522	−0.094	−0.441	−0.267	0.066	0.242	0.694
	1995		−0.139	−0.318	0.154	−0.339	0.439	0.637	0.148	0.034	0.440	0.336	0.548	0.533	−0.089	−0.427	−0.269	0.042	0.275	0.674
	2000		−0.209	−0.366	0.155	−0.339	0.439	0.619	0.136	0.040	0.440	0.334	0.543	0.543	−0.083	−0.399	−0.272	0.005	0.268	0.671
	2005		−0.240	−0.357	0.200	−0.330	0.434	0.600	0.140	0.041	0.445	0.318	0.530	0.529	−0.079	−0.399	−0.284	0.015	0.239	0.661
	2010		−0.255	−0.382	0.212	−0.370	0.437	0.595	0.136	0.038	0.427	0.322	0.545	0.532	−0.086	−0.394	−0.272	0.055	0.212	0.657
	2015		−0.281	−0.393	0.199	−0.355	0.432	0.584	0.130	0.031	0.427	0.322	0.546	0.530	−0.081	−0.400	−0.281	0.092	0.202	0.652
	2017		−0.280	−0.397	0.196	−0.364	0.429	0.582	0.130	0.032	0.429	0.323	0.544	0.528	−0.073	−0.393	−0.282	0.114	0.190	0.650
MSK	1990	Males	0.258	0.084	−0.026	−0.277	0.500	0.482	0.175	0.146	0.198	0.145	0.522	0.366	0.243	−0.250	0.215	0.083	0.195	0.384
	1995		0.218	−0.022	−0.045	−0.356	0.485	0.467	0.154	0.127	0.178	0.161	0.523	0.364	0.236	−0.248	0.223	0.050	0.218	0.376
	2000		0.195	−0.115	−0.037	−0.333	0.479	0.440	0.151	0.128	0.146	0.169	0.524	0.363	0.257	−0.213	0.236	0.021	0.202	0.338
	2005		0.133	−0.155	−0.033	−0.354	0.463	0.431	0.139	0.111	0.158	0.153	0.519	0.353	0.252	−0.219	0.263	0.044	0.191	0.324
	2010		0.099	−0.193	−0.004	−0.420	0.449	0.424	0.119	0.093	0.174	0.150	0.525	0.339	0.254	−0.221	0.275	0.053	0.172	0.308
	2015		0.015	−0.230	−0.010	−0.400	0.440	0.422	0.108	0.082	0.172	0.150	0.516	0.336	0.272	−0.229	0.288	0.073	0.155	0.286
	2017		0.004	−0.245	−0.024	−0.426	0.438	0.423	0.107	0.079	0.169	0.154	0.513	0.334	0.281	−0.223	0.287	0.082	0.146	0.277

All significant correlations (*p* < 0.05) are in red. MSK is the sum of DALYs due to rheumatoid arthritis, osteoarthritis, low back pain, neck pain, gout and other MSK. DALYs: Disability-adjusted life-years; CVD: Cardiovascular disorders; NeuroD: Neurological Disorders; MDT: Total Mental Disorders; aMeAD: All Mental except Anxiety and Depressive disorders; Mental MDD: Major Depressive Disorders; ADHD: Attention deficit/hyperactivity disorder; IDID: Idiopathic Developmental Intellectual disability; EatingD: Anorexia or Bulimia nervosa; OMD; Other Mental Disorders; SZA: Schizophrenia; MSK: Musculoskeletal Disorders.

**Table 4:** Spearman’s correlation coefficients between age-standardized DALYs attributed to musculoskeletal disorders and age-standardized DALYs attributed to various disorders, among 195 countries, in both sexes, by country’s income categorization for 1990 to 2017.

			Other Disorders				Mental Disorders Sub-Categories													
	Year	Income Categories	Neoplasms	CVD	NeuroD	Injuries	MDT	aMeAD	Depressive Disorders	MDD	Dysthymia	Bipolar	Anxiety	EatingD	Autism Disorders	ADHD	Conduct Disorder	IDID	OMD	SZA
MSK	1990	Low income	−0.401	0.120	−0.035	−0.065	0.162	0.375	−0.062	−0.063	−0.404	0.299	0.201	0.442	−0.414	−0.293	0.224	0.095	0.092	0.490
	1995		−0.490	−0.024	−0.191	−0.236	0.085	0.368	−0.127	−0.128	−0.450	0.315	0.162	0.426	−0.365	0.243	0.273	0.073	0.151	0.509
	2000		−0.422	0.013	−0.063	−0.208	0.066	0.494	−0.096	−0.097	−0.446	0.340	0.163	0.417	−0.348	−0.234	0.260	0.093	0.229	0.569
	2005		−0.291	0.151	0.191	−0.099	0.058	0.522	−0.075	−0.083	−0.373	0.316	0.097	0.399	−0.347	−0.283	0.238	0.203	0.195	0.547
	2010		−0.315	0.187	0.275	−0.201	0.088	0.465	−0.033	−0.062	−0.397	0.274	0.061	0.414	−0.377	−0.338	0.182	0.155	0.054	0.524
	2015		−0.364	0.157	0.332	0.032	0.058	0.462	−0.063	−0.064	−0.360	0.246	0.001	0.365	−0.396	−0.394	0.210	0.185	0.007	0.497
	2017		−0.392	0.128	0.327	−0.082	0.029	0.445	−0.051	−0.059	−0.334	0.234	−0.014	0.330	−0.356	−0.378	0.230	0.149	−0.035	0.502
MSK	1990	Lower and upper-middle income	0.037	0.400	0.192	−0.034	0.175	0.214	0.064	−0.035	0.273	−0.171	0.333	−0.263	−0.541	−0.259	0.026	0.561	−0.002	0.333
	1995		−0.007	0.339	0.180	−0.084	0.143	0.189	0.037	−0.045	0.237	−0.148	0.323	−0.230	−0.524	−0.254	0.003	0.582	0.084	0.314
	2000		−0.056	0.247	0.147	−0.091	0.139	0.162	0.039	−0.046	0.203	−0.124	0.319	−0.194	−0.500	−0.213	0.005	0.559	0.071	0.299
	2005		−0.085	0.232	0.118	−0.133	0.115	0.123	0.022	−0.056	0.212	−0.129	0.316	−0.201	−0.499	−0.229	0.007	0.562	0.051	0.308
	2010		−0.067	0.221	0.143	−0.199	0.108	0.120	0.009	−0.067	0.226	−0.117	0.323	−0.210	−0.508	−0.235	−0.005	0.578	0.042	0.305
	2015		−0.083	0.195	0.105	−0.186	0.103	0.101	−0.009	−0.075	0.226	−0.105	0.333	−0.217	−0.507	−0.238	0.002	0.595	0.047	0.291
	2017		−0.091	0.205	0.111	−0.191	0.101	0.082	−0.007	−0.079	0.223	−0.103	0.327	−0.232	−0.510	−0.240	−0.003	0.587	0.033	0.272
MSK	1990	High income	0.111	−0.457	0.418	−0.288	0.735	0.719	0.506	0.376	0.504	0.345	0.656	0.652	0.670	−0.241	0.061	0.214	0.565	0.391
	1995		0.083	−0.514	0.390	−0.313	0.750	0.694	0.516	0.413	0.492	0.344	0.643	0.630	0.651	−0.233	0.121	0.202	0.523	0.373
	2000		−0.042	−0.488	0.380	−0.321	0.741	0.680	0.521	0.421	0.462	0.331	0.642	0.623	0.637	−0.238	0.142	0.184	0.505	0.355
	2005		−0.116	−0.550	0.373	−0.415	0.723	0.661	0.567	0.453	0.480	0.316	0.625	0.594	0.643	−0.235	0.154	0.173	0.493	0.339
	2010		−0.151	−0.568	0.385	−0.447	0.726	0.648	0.566	0.470	0.478	0.304	0.650	0.582	0.640	−0.235	0.146	0.140	0.499	0.325
	2015		−0.206	−0.592	0.372	−0.453	0.723	0.658	0.564	0.456	0.495	0.316	0.649	0.608	0.660	−0.224	0.157	0.173	0.482	0.328
	2017		−0.206	−0.594	0.384	−0.485	0.727	0.670	0.566	0.437	0.496	0.317	0.667	0.608	0.669	−0.230	0.135	0.207	0.498	0.329

All significant correlations (*p* < 0.05) are in red. Country Income categorization is adopted the World Bank. DALYs: Disability-adjusted life-years; CVD: Cardiovascular disorders; NeuroD: Neurological Disorders; MDT: Total Mental Disorders; aMeAD: All Mental except Anxiety and Depressive disorders; Mental MDD: Major Depressive Disorders; ADHD: Attention deficit/hyperactivity disorder; IDID: Idiopathic Developmental Intellectual disability; EatingD: Anorexia or Bulimia nervosa; OMD; Other Mental Disorder; SZA: Schizophrenia; MSK: Musculoskeletal Disorder. MSK is the sum of DALYs due to rheumatoid arthritis; osteoarthritis; low back pain; neck pain; gout and other MSK disorders.

## Supplementary Materials

The following are available online at https://www.mdpi.com/2077-0383/9/7/2189/s1, Table S1: Mental and musculoskeletal disorders age-standardised DALYs ARC% estimates for both sexes, between 1990 and 2017. Table S2: Spearman’s correlation coefficients between age-standardized DALYs attributed to total mental disorders (MDT) and age-standardized DALYs attributed to various disorder groups, among 195 countries, in both sexes, for 1990 to 2017. Table S3: Spearman’s correlation coefficients between age-standardized DALYs attributed to total mental disorders (MDT) and age-standardized DALYs attributed to various disorders, among 195 countries, in males and females, for 1990 to 2017. Table S4: Spearman’s correlation coefficients between age-standardized DALYs attributed to total mental disorders (MDT) and age-standardized DALYs attributed to various disorders, among 195 countries, in both sexes, by country’s income categorization for 1990 to 2017. Table S5. Spearman correlations between age-standardized YLDs attributed to musculoskeletal disorders and age-standardized YLDs attributed to various disorders, among 195 countries, in both sexes, for 1990 to 2017. Table S6. Spearman correlations between age-standardized YLDs attributed to musculoskeletal disorders and age-standardized YLDs attributed to various disorders. among 195 countries, in males and females, for 1990 to 2017. Table S7. Spearman correlations between age-standardized YLDs attributed to musculoskeletal disorders and age-standardized YLDs attributed to various disorders, among 195 countries, in both sexes, by country’s income categorization for 1990 to 2017.
