# Supplementary material for: Mental Disorders, Musculoskeletal Disorders and Income-Driven Patterns: Evidence from the Global Burden of Disease Study 2017
Source: J Clin Med. 2020 Jul 10;9(7):2189. doi: 10.3390/jcm9072189 (PMC7408666; doi:10.3390/jcm9072189)
Supplement: Supplementary file 1 [file jcm-09-02189-s001.pdf]

Supplementary Materials:

Mental Disorders, Musculoskeletal Disorders and Income-Driven Patterns: Evidence from the Global Burden of Disease Study 2017

Table of Contents

Supplementary Table S1. ………………………………………………….......................2
Supplementary Table S2. ……………………………………………………………………7
Supplementary Table S3. ……………………………………………………………………8
Supplementary Table S4…………….………………………………………………………..9
Supplementary Table S5…………….………………………………………………………..10
Supplementary Table S6…………….………………………………………………………..11
Supplementary Table S7…………….………………………………………………………..12

Supplementary Table S1. Mental and musculoskeletal disorders age-standardised DALYs ARC% estimates for both sexes, between 1990 and 2017

Location	DALYs ARC % 1990-2017	
	**Mental Disorders**	**Musculoskeletal Disorders**
Mozambique	0.023%	0.237%
Mauritius	-0.146%	0.168%
Malawi	-0.053%	0.190%
Rwanda	-0.192%	-0.311%
Somalia	0.025%	0.160%
Zambia	0.037%	0.192%
Kenya	-0.074%	0.055%
Madagascar	-0.061%	-0.101%
South Sudan	0.049%	0.086%
Seychelles	-0.071%	0.053%
Tanzania	-0.049%	-0.022%
Uganda	0.012%	0.202%
Zimbabwe	0.047%	0.156%
Ethiopia	-0.139%	0.083%
Eritrea	-0.015%	0.072%
Djibouti	0.004%	0.189%
Comoros	-0.023%	0.247%
Burundi	-0.247%	-0.131%
Gabon	-0.029%	0.106%
Equatorial Guinea	0.044%	0.106%
Chad	0.028%	0.310%
Sao Tome and Principe	-0.019%	0.256%
Congo	-0.071%	-0.004%
Democratic Republic of the Congo	-0.018%	-0.002%
Cameroon	0.087%	0.304%
Central African Republic	0.019%	-0.018%
Angola	-0.013%	0.056%
Morocco	-0.058%	0.268%
Libya	0.010%	0.060%
Tunisia	-0.029%	0.078%
Algeria	-0.061%	0.121%
Namibia	0.004%	0.099%
Swaziland	0.109%	-0.109%
Lesotho	0.090%	-0.020%
South Africa	-0.034%	-0.273%
Botswana	0.065%	0.065%
Togo	0.061%	0.282%
Sierra Leone	0.078%	0.192%
Senegal	0.034%	0.250%
Nigeria	-0.013%	0.129%

Niger	0.046%	0.260%
Mauritania	-0.038%	0.287%
Mali	-0.017%	0.222%
Liberia	0.126%	0.020%
Guinea-Bissau	0.058%	0.272%
The Gambia	-0.005%	0.268%
Guinea	0.055%	0.270%
Ghana	0.010%	-0.012%
Cape Verde	0.132%	0.266%
Cote d'Ivoire	0.055%	0.347%
Burkina Faso	0.069%	0.358%
Benin	0.075%	0.371%
Dominica	0.006%	0.064%
Dominican Republic	0.040%	0.031%
Antigua and Barbuda	0.002%	-0.007%
Cuba	-0.338%	-0.153%
Grenada	0.025%	-0.129%
Jamaica	-0.007%	-0.159%
Trinidad and Tobago	-0.025%	-0.037%
The Bahamas	-0.025%	-0.162%
Saint Lucia	0.024%	-0.124%
Saint Vincent and the Grenadines	0.055%	0.138%
Barbados	0.008%	-0.062%
Haiti	-0.003%	-0.312%
Puerto Rico	-0.008%	0.004%
Virgin Islands. U.S.	0.091%	-0.030%
Panama	0.035%	0.052%
El Salvador	-0.061%	0.250%
Nicaragua	0.040%	0.160%
Mexico	0.111%	-0.043%
Honduras	0.055%	0.187%
Guatemala	-0.010%	0.340%
Costa Rica	0.078%	0.045%
Belize	0.065%	0.076%
United States	0.084%	0.039%
Greenland	0.018%	0.157%
Bermuda	-0.102%	-0.124%
Colombia	-0.072%	0.309%
Guyana	0.198%	0.055%
Ecuador	0.052%	0.227%
Paraguay	0.088%	0.413%
Suriname	0.134%	0.032%
Uruguay	0.141%	0.115%
Venezuela	0.021%	-0.111%

Peru	-0.015%	0.158%
Chile	-0.137%	0.424%
Brazil	-0.008%	0.001%
Bolivia	-0.014%	0.170%
Argentina	0.034%	0.082%
Turkmenistan	-0.062%	-0.026%
Uzbekistan	-0.054%	-0.077%
Tajikistan	-0.090%	-0.069%
Kyrgyzstan	-0.084%	-0.023%
Kazakhstan	0.063%	-0.044%
Mongolia	0.074%	-0.130%
Taiwan	0.107%	0.046%
South Korea	0.193%	0.099%
North Korea	-0.049%	-0.050%
Japan	0.136%	0.133%
Myanmar	-0.164%	-0.020%
Cambodia	-0.214%	0.028%
Vietnam	-0.145%	0.127%
Laos	-0.126%	0.077%
Malaysia	0.081%	-0.025%
Thailand	-0.111%	-0.319%
Brunei	-0.028%	0.112%
Philippines	-0.196%	0.044%
Indonesia	-0.113%	0.145%
Singapore	-0.180%	-0.251%
Timor-Leste	-0.250%	-0.032%
Maldives	-0.221%	-0.062%
Nepal	-0.046%	0.207%
Iran	0.085%	0.112%
Sri Lanka	-0.263%	-0.029%
Bhutan	-0.089%	-0.035%
Bangladesh	-0.108%	-0.154%
Afghanistan	-0.030%	0.139%
Oman	-0.056%	0.087%
Kuwait	0.061%	0.205%
Palestine	-0.084%	-0.040%
Lebanon	0.115%	0.149%
Qatar	-0.095%	0.072%
Saudi Arabia	0.029%	0.176%
Turkey	-0.040%	-0.084%
Yemen	0.053%	0.073%
Iraq	0.037%	0.041%
Jordan	-0.140%	-0.113%
Syria	-0.080%	-0.116%

Israel	-0.062%	-0.074%
Georgia	-0.012%	0.098%
Cyprus	-0.005%	-0.023%
Bahrain	-0.146%	0.036%
Azerbaijan	-0.006%	0.047%
Armenia	0.079%	0.138%
United Arab Emirates	-0.112%	0.068%
Moldova	-0.165%	-0.073%
Hungary	-0.211%	-0.007%
Poland	0.023%	0.016%
Romania	0.018%	-0.048%
Ukraine	-0.142%	0.025%
Belarus	0.014%	0.046%
Czech Republic	-0.131%	-0.052%
Russian Federation	-0.028%	-0.324%
Slovakia	-0.052%	0.154%
Bulgaria	-0.118%	-0.056%
Norway	-0.072%	-0.137%
Sweden	-0.059%	-0.098%
Lithuania	-0.052%	0.183%
Latvia	-0.240%	0.231%
Ireland	0.095%	-0.117%
Iceland	-0.062%	-0.289%
United Kingdom	-0.054%	0.222%
Finland	-0.230%	-0.355%
Estonia	-0.322%	0.152%
Denmark	-0.227%	0.221%
Macedonia	-0.051%	0.013%
Montenegro	0.003%	0.041%
Croatia	-0.179%	0.177%
Malta	-0.022%	0.001%
Slovenia	-0.252%	0.106%
Greece	0.020%	0.009%
Italy	-0.150%	-0.085%
Portugal	-0.109%	0.095%
Serbia	-0.092%	0.002%
Spain	-0.047%	-0.121%
Bosnia and Herzegovina	-0.219%	0.040%
Andorra	-0.010%	0.028%
Albania	0.033%	0.019%
Netherlands	0.015%	-0.210%
Luxembourg	-0.123%	0.001%
France	-0.112%	0.123%
Germany	0.018%	-0.054%

Switzerland	-0.192%	0.363%
Belgium	0.207%	-0.029%
Austria	-0.102%	0.022%
New Zealand	-0.023%	0.044%
Australia	0.058%	-0.057%
Papua New Guinea	-0.032%	0.078%
Vanuatu	-0.031%	0.053%
Solomon Islands	-0.035%	0.073%
Fiji	0.024%	0.068%
Guam	0.038%	0.058%
Northern Mariana Islands	0.032%	-0.007%
Marshall Islands	-0.033%	0.027%
Federated States of Micronesia	-0.044%	0.050%
Kiribati	-0.087%	0.145%
Samoa	-0.075%	-0.054%
Tonga	-0.024%	0.002%
American Samoa	-0.041%	0.044%
Canada	-0.059%	-0.038%
Egypt	-0.029%	0.049%
Sudan	-0.076%	0.078%
India	-0.155%	-0.153%
Pakistan	-0.105%	0.115%
China	-0.144%	-0.278%

DALYs: Disability-adjusted life-years
ARC %: Annualized rate of change %
Table S1 was generated from the GBD compare visualization tool at https://vizhub.healthdata.org/gbd-compare/

Supplementary Table S2. Spearman's correlation coefficients between age-standardized DALYs attributed to total mental disorders (MDT) and age-standardized DALYs attributed to various disorder groups, among 195 countries, in both sexes, for 1990 to 2017.

		Other Disorders				Musculoskeletal Disorders sub-categories						
	**Year**	**Neoplasm**	**CVD**	**NeuroD**	**Injuries**	**MSK**	**Rheumatoid arthistis**	**Osteoarthritis**	**Low back pain**	**Neck pain**	**Gout**	**Other MSK**
MDT	1990	0.004	-0.233	0.351	-0.128	0.487	0.484	0.446	0.375	0.262	0.353	0.167
	1995	-0.019	-0.296	0.297	-0.199	0.470	0.479	0.431	0.361	0.274	0.352	0.165
	2000	-0.052	-0.332	0.279	-0.145	0.462	0.492	0.418	0.359	0.260	0.342	0.161
	2005	-0.148	-0.382	0.249	-0.153	0.440	0.501	0.400	0.337	0.254	0.336	0.166
	2010	-0.193	-0.407	0.274	-0.185	0.441	0.511	0.407	0.322	0.261	0.341	0.172
	2015	-0.243	-0.417	0.268	-0.183	0.441	0.532	0.417	0.320	0.268	0.358	0.164
	2017	-0.254	-0.423	0.265	-0.193	0.439	0.541	0.420	0.316	0.271	0.366	0.160

All significant correlations ($p<0.05$) are in red.

CVD: Cardiovascular disorders; NeuroD: Neurological Disorders; MDT: Total Mental Disorders; MSK: Musculoskeletal Disorders; Other MSK: Other Musculoskeletal Disorders; DALYs: Disability-adjusted life-years

Supplementary Table S3. Spearman's correlation coefficients between age-standardized DALYs attributed to total mental disorders (MDT) and age-standardized DALYs attributed to various disorders, among 195 countries, in males and females, for 1990 to 2017.

			Other Disorders				Musculoskeletal Disorders sub-categories						
	Year	Gender	Neoplasm	CVD	NeuroD	Injuries	MSK	Rheumatoid arthistis	Osteo arthritis	Low back pain	Neck pain	Gout	Other MSK
MDT	1990	Males	-0.075	-0.144	0.187	-0.141	0.500	0.311	0.423	0.280	0.241	0.325	0.244
	1995		-0.063	-0.200	0.123	-0.215	0.485	0.300	0.417	0.269	0.261	0.329	0.240
	2000		-0.067	-0.263	0.124	-0.117	0.479	0.331	0.432	0.252	0.240	0.330	0.232
	2005		-0.148	-0.312	0.091	-0.121	0.463	0.349	0.423	0.229	0.235	0.329	0.240
	2010		-0.182	-0.341	0.108	-0.158	0.449	0.362	0.408	0.208	0.248	0.327	0.259
	2015		-0.230	-0.361	0.090	-0.146	0.440	0.374	0.418	0.195	0.254	0.337	0.260
	2017		-0.235	-0.365	0.087	-0.155	0.438	0.383	0.429	0.192	0.255	0.344	0.256
MDT	1990	Females	0.024	-0.258	0.438	-0.071	0.448	0.539	0.437	0.351	0.261	0.219	0.151
	1995		0.007	-0.298	0.420	-0.131	0.439	0.545	0.413	0.342	0.262	0.213	0.139
	2000		-0.028	-0.341	0.414	-0.113	0.439	0.562	0.396	0.350	0.253	0.213	0.134
	2005		-0.128	-0.383	0.384	-0.136	0.434	0.570	0.386	0.342	0.248	0.227	0.139
	2010		-0.165	-0.402	0.392	-0.153	0.437	0.587	0.394	0.327	0.260	0.277	0.136
	2015		-0.202	-0.411	0.393	-0.153	0.432	0.613	0.391	0.328	0.267	0.279	0.117
	2017		-0.185	-0.414	0.396	-0.160	0.429	0.629	0.390	0.327	0.268	0.284	0.106

All significant correlations ($p<0.05$) are in red.

CVD: Cardiovascular disorders; NeuroD: Neurological Disorders; MDT: Total Mental Disorders; MSK: Musculoskeletal Disorders; Other MSK: Other Musculoskeletal Disorders; DALYs: Disability-adjusted life-years

Supplementary Table S4. Spearman's correlation coefficients between age-standardized DALYs attributed to total mental disorders (MDT) and age-standardized DALYs attributed to various disorders, among 195 countries, in both sexes, by country´s income categorization for 1990 to 2017.

			Other Disorders				Musculoskeletal Disorders sub-categories						
	**Year**	**Income categories**	**Neoplasm**	**CVD**	**NeuroD**	**Injuries**	**MSK**	**Rheumatoid arthistis**	**Osteo arthritis**	**Low back pain**	**Neck pain**	**Gout**	**Other MSK**
MDT	1990	Low income	-0.032	0.474	0.155	0.157	0.162	0.461	0.273	0.250	-0.125	-0.224	-0.037
	1995		0.108	0.505	0.168	0.079	0.085	0.464	0.212	0.198	-0.150	-0.315	-0.034
	2000		0.087	0.394	-0.079	0.319	0.066	0.497	0.221	0.199	-0.147	-0.278	-0.006
	2005		-0.118	0.242	-0.167	0.396	0.058	0.468	0.156	0.166	-0.098	-0.347	-0.009
	2010		-0.115	0.168	-0.156	0.184	0.088	0.482	0.141	0.166	-0.108	-0.301	0.062
	2015		-0.164	0.157	-0.182	0.351	0.058	0.477	0.141	0.145	-0.104	-0.315	0.064
	2017		-0.162	0.114	-0.203	0.269	0.029	0.482	0.141	0.126	-0.111	-0.328	0.045
MDT	1990	Lower & upper-middle income	-0.246	-0.108	0.425	0.141	0.175	0.429	0.232	0.061	-0.091	0.040	0.136
	1995		-0.247	-0.165	0.351	0.110	0.143	0.425	0.225	0.047	-0.056	0.045	0.136
	2000		-0.190	-0.202	0.366	0.146	0.139	0.437	0.194	0.053	-0.098	0.009	0.107
	2005		-0.252	-0.246	0.310	0.162	0.115	0.445	0.195	0.025	-0.110	-0.005	0.126
	2010		-0.266	-0.257	0.335	0.140	0.108	0.467	0.199	0.008	-0.106	-0.013	0.119
	2015		-0.273	-0.261	0.350	0.129	0.103	0.503	0.213	-0.001	-0.100	-0.006	0.106
	2017		-0.289	-0.263	0.347	0.106	0.101	0.516	0.222	-0.007	-0.097	0.005	0.093
MDT	1990	High income	0.057	-0.446	0.447	-0.206	0.735	0.441	0.440	0.436	0.482	0.578	0.004
	1995		0.026	-0.492	0.440	-0.311	0.750	0.460	0.434	0.392	0.491	0.593	0.013
	2000		-0.116	-0.467	0.448	-0.309	0.741	0.508	0.454	0.378	0.501	0.609	0.042
	2005		-0.203	-0.536	0.428	-0.390	0.723	0.528	0.446	0.365	0.487	0.626	0.028
	2010		-0.243	-0.569	0.463	-0.405	0.726	0.528	0.474	0.349	0.476	0.647	0.058
	2015		-0.284	-0.588	0.453	-0.401	0.723	0.523	0.475	0.332	0.479	0.654	0.058
	2017		-0.289	-0.584	0.482	-0.401	0.727	0.536	0.480	0.322	0.489	0.658	0.067

All significant correlations (p<0.05) are in red.

CVD: Cardiovascular disorders; NeuroD: Neurological Disorders; MDT: Total Mental Disorders; MSK: Musculoskeletal Disorders; Other MSK: Other Musculoskeletal Disorders; DALYs: Disability-adjusted life-years

Supplementary Table S5. Spearman correlations between age-standardized YLDs attributed to musculoskeletal disorders and age-standardized YLDs attributed to various disorders, among 195 countries, in both sexes, for 1990 to 2017.

		Other Disorders				Mental Disorders sub-categories													
	**Year**	**Neoplasms**	**CVD**	**NeuroD**	**Injuries**	**MDT**	**aMeAD**	**Depressive disorders**	**MDD**	**Dysthymia**	**Bipolar**	**Anxiety**	**EatingD**	**Autism disorders**	**ADHD**	**Conduct disorder**	**IDID**	**OMD**	**SZA**
MSK	1990	0.366	0.498	0.298	0.227	0.464	0.626	0.132	0.038	0.341	0.277	0.523	0.454	0.136	-0.321	0.028	0.083	0.465	0.601
	1995	0.351	0.439	0.313	0.221	0.449	0.609	0.111	0.028	0.326	0.282	0.504	0.460	0.148	-0.323	0.046	0.056	0.489	0.579
	2000	0.360	0.357	0.340	0.201	0.444	0.586	0.121	0.037	0.308	0.280	0.496	0.464	0.162	-0.299	0.043	0.027	0.481	0.563
	2005	0.375	0.309	0.354	0.228	0.427	0.569	0.114	0.032	0.317	0.267	0.488	0.453	0.168	-0.306	0.067	0.040	0.466	0.552
	2010	0.401	0.249	0.342	0.212	0.428	0.566	0.107	0.026	0.321	0.266	0.492	0.454	0.167	-0.309	0.054	0.048	0.448	0.542
	2015	0.405	0.218	0.328	0.251	0.427	0.559	0.106	0.022	0.331	0.264	0.484	0.447	0.176	-0.318	0.058	0.086	0.441	0.531
	2017	0.404	0.203	0.325	0.258	0.425	0.555	0.105	0.016	0.332	0.261	0.481	0.443	0.182	-0.321	0.054	0.098	0.439	0.527

All significant correlations ($p<0.05$) are in red.

CVD: Cardiovascular disorders; NeuroD: Neurological Disorders; MDT: Total Mental Disorders; aMeAD: All Mental except Anxiety and Depressive disorders; Mental MDD: Major Depressive Disorders; ADHD: Attention deficit/hyperactivity disorder; IDID: Idiopathic Developmental Intellectual disability; EatingD: Anorexia or Bulimia nervosa; OMD; Other Mental Disorder; SZA: Schizophrenia; MSK: Musculoskeletal Disorders; YLDs: Years lived with Disability

Supplementary Table S6. Spearman correlations between age-standardized YLDs attributed to musculoskeletal disorders and age-standardized YLDs attributed to various disorders. among 195 countries, in males and females, for 1990 to 2017.

			Other Disorders				Mental Disorders sub-categories													
	**Year**	**Gender**	**Neoplasms**	**CVD**	**NeuroD**	**Injuries**	**MDT**	**aMeAD**	**Depressive disorders**	**MDD**	**Dysthymia**	**Bipolar**	**Anxiety**	**EatingD**	**Autism disorders**	**ADHD**	**Conduct disorder**	**IDID**	**OMD**	**SZA**
	1990		0.347	0.439	0.365	0.193	0.437	0.634	0.178	0.059	0.469	0.318	0.534	0.502	-0.081	-0.455	-0.244	0.084	0.232	0.696
	1995		0.356	0.374	0.369	0.199	0.425	0.627	0.151	0.035	0.466	0.323	0.527	0.509	-0.078	-0.447	-0.240	0.066	0.267	0.676
MSK	2000		0.390	0.291	0.377	0.182	0.424	0.606	0.140	0.041	0.470	0.317	0.519	0.515	-0.072	-0.422	-0.239	0.034	0.264	0.673
	2005		0.417	0.226	0.389	0.204	0.415	0.588	0.141	0.037	0.472	0.302	0.506	0.505	-0.064	-0.420	-0.251	0.040	0.235	0.665
	2010		0.449	0.159	0.381	0.173	0.419	0.580	0.141	0.038	0.458	0.303	0.519	0.511	-0.065	-0.413	-0.238	0.074	0.203	0.661
	2015	Female	0.449	0.130	0.375	0.178	0.415	0.572	0.135	0.032	0.458	0.304	0.519	0.512	-0.058	-0.418	-0.244	0.106	0.194	0.658
	2017		0.456	0.117	0.375	0.179	0.412	0.570	0.135	0.034	0.457	0.308	0.518	0.510	-0.048	-0.412	-0.244	0.126	0.184	0.657
	1990		0.356	0.510	0.177	0.269	0.477	0.495	0.143	0.114	0.183	0.170	0.522	0.387	0.236	-0.263	0.226	0.073	0.208	0.399
	1995		0.327	0.467	0.205	0.243	0.467	0.487	0.121	0.096	0.161	0.187	0.524	0.387	0.223	-0.264	0.229	0.044	0.238	0.398
MSK	2000		0.325	0.396	0.216	0.238	0.455	0.461	0.113	0.091	0.137	0.190	0.523	0.383	0.248	-0.234	0.240	0.019	0.228	0.361
	2005		0.325	0.370	0.228	0.261	0.444	0.452	0.106	0.081	0.141	0.180	0.519	0.375	0.249	-0.228	0.274	0.039	0.212	0.338
	2010		0.342	0.340	0.221	0.251	0.434	0.439	0.093	0.069	0.160	0.168	0.526	0.360	0.253	-0.234	0.287	0.042	0.188	0.320
	2015	Male	0.338	0.308	0.225	0.309	0.430	0.434	0.089	0.065	0.159	0.168	0.520	0.351	0.271	-0.236	0.299	0.061	0.162	0.293
	2017		0.342	0.293	0.227	0.327	0.425	0.436	0.086	0.060	0.157	0.169	0.512	0.352	0.282	-0.234	0.298	0.071	0.153	0.286

All significant correlations ($p<0.05$) are in red.

CVD: Cardiovascular disorders; NeuroD: Neurological Disorders; MDT: Total Mental Disorders; aMeAD: All Mental except Anxiety and Depressive disorders; Mental MDD: Major Depressive Disorders; ADHD: Attention deficit/hyperactivity disorder; IDID: Idiopathic Developmental Intellectual disability; EatingD: Anorexia or Bulimia nervosa; OMD; Other Mental Disorder; SZA: Schizophrenia; MSK: Musculoskeletal Disorders; YLDs: Years lived with Disability

Supplementary Table S7. Spearman correlations between age-standardized YLDs attributed to musculoskeletal disorders and age-standardized YLDs attributed to various disorders, among 195 countries, in both sexes, by country´s income categorization for 1990 to 2017.

			Other Disorders				Mental Disorders sub-categories													
	**Year**	**Income Categories**	**Neoplasms**	**CVD**	**NeuroD**	**Injuries**	**MDT**	**aMeAD**	**Depressive disorders**	**MDD**	**Dysthymia**	**Bipolar**	**Anxiety**	**EatingD**	**Autism disorders**	**ADHD**	**Conduct disorder**	**IDID**	**OMD**	**SZA**
MSK	1990	Low income	-0.515	0.281	0.657	-0.326	0.083	0.331	-0.114	-0.114	-0.431	0.297	0.106	0.452	-0.417	-0.340	0.208	0.052	0.078	0.509
	1995		-0.549	0.326	0.660	-0.390	0.031	0.388	-0.173	-0.172	-0.436	0.357	0.102	0.431	-0.353	-0.250	0.305	0.070	0.175	0.552
	2000		-0.445	0.361	0.669	-0.425	0.018	0.458	-0.098	-0.100	-0.396	0.339	0.082	0.418	-0.342	-0.284	0.296	0.072	0.203	0.582
	2005		-0.289	0.426	0.693	-0.417	0.016	0.506	-0.080	-0.089	-0.368	0.304	0.016	0.379	-0.343	-0.332	0.264	0.204	0.201	0.556
	2010		-0.263	0.447	0.707	-0.459	0.067	0.458	-0.036	-0.066	-0.405	0.269	0.018	0.406	-0.368	-0.369	0.212	0.160	0.036	0.525
	2015		-0.227	0.446	0.687	-0.325	0.030	0.466	-0.071	-0.073	-0.351	0.254	-0.037	0.359	-0.363	-0.409	0.249	0.177	0.029	0.507
	2017		-0.259	0.382	0.679	-0.357	0.009	0.447	-0.049	-0.058	-0.327	0.238	-0.056	0.330	-0.333	-0.397	0.275	0.142	-0.037	0.512
MSK	1990	Lower & upper-middle income	-0.033	0.569	0.114	0.110	0.142	0.219	0.040	-0.057	0.267	-0.155	0.301	-0.262	-0.517	-0.267	0.071	0.571	-0.019	0.330
	1995		-0.062	0.544	0.140	0.108	0.119	0.188	0.028	-0.056	0.249	-0.146	0.287	-0.244	-0.502	-0.274	0.052	0.604	0.061	0.308
	2000		-0.089	0.511	0.187	0.082	0.109	0.163	0.026	-0.059	0.212	-0.125	0.275	-0.215	-0.470	-0.238	0.064	0.587	0.049	0.289
	2005		-0.090	0.509	0.191	0.064	0.095	0.124	0.021	-0.062	0.228	-0.131	0.278	-0.224	-0.466	-0.251	0.062	0.590	0.020	0.293
	2010		-0.035	0.477	0.162	0.051	0.084	0.125	0.000	-0.081	0.242	-0.116	0.291	-0.223	-0.480	-0.250	0.051	0.600	0.017	0.298
	2015		-0.043	0.474	0.155	0.116	0.067	0.088	-0.016	-0.089	0.250	-0.118	0.284	-0.239	-0.473	-0.263	0.071	0.615	0.008	0.274
	2017		-0.039	0.471	0.156	0.139	0.073	0.066	-0.011	-0.090	0.251	-0.120	0.283	-0.250	-0.477	-0.266	0.059	0.603	0.002	0.259
	1990	High income	0.610	-0.220	0.450	0.290	0.724	0.702	0.505	0.375	0.514	0.344	0.641	0.637	0.652	-0.257	0.062	0.234	0.568	0.396
	1995		0.583	-0.280	0.423	0.308	0.740	0.682	0.506	0.403	0.493	0.337	0.637	0.612	0.647	-0.241	0.120	0.206	0.526	0.372
	2000		0.584	-0.342	0.450	0.312	0.733	0.677	0.513	0.411	0.467	0.335	0.639	0.621	0.634	-0.250	0.135	0.195	0.509	0.354
	2005		0.579	-0.361	0.460	0.326	0.720	0.655	0.562	0.447	0.487	0.313	0.624	0.591	0.637	-0.244	0.152	0.183	0.502	0.343

MSK	2010	0.545	-0.385	0.466	0.331	0.721	0.647	0.564	0.466	0.482	0.310	0.644	0.586	0.634	-0.246	0.133	0.140	0.501	0.331
	2015	0.528	-0.433	0.458	0.337	0.722	0.660	0.564	0.453	0.503	0.318	0.646	0.612	0.659	-0.233	0.146	0.178	0.490	0.330
	2017	0.534	-0.436	0.450	0.335	0.722	0.662	0.564	0.433	0.501	0.314	0.665	0.612	0.663	-0.237	0.126	0.209	0.504	0.329

All significant correlations ($p<0.05$) are in red.
Country Income categorization is adopted the World Bank
CVD: Cardiovascular disorders; NeuroD: Neurological Disorders; MDT: Total Mental Disorders; aMeAD: All Mental except Anxiety and Depressive disorders; Mental MDD: Major Depressive Disorders; ADHD: Attention deficit/hyperactivity disorder; IDID: Idiopathic Developmental Intellectual disability; EatingD: Anorexia or Bulimia nervosa; OMD; Other Mental Disorder; SZA: Schizophrenia; MSK: Musculoskeletal Disorders; YLDs: Years lived with Disability
